# Collagen type II (CII)-specific antibodies induce arthritis in the absence of T or B cells but the arthritis progression is enhanced by CII-reactive T cells

**DOI:** 10.1186/ar1217

**Published:** 2004-09-23

**Authors:** Kutty Selva Nandakumar, Johan Bäcklund, Mikael Vestberg, Rikard Holmdahl

**Affiliations:** 1 Section for Medical Inflammation Research, Lund University, Sweden

**Keywords:** arthritis, B cells, collagen type II, monoclonal antibodies, T cells

## Abstract

Antibodies against type II collagen (anti-CII) are arthritogenic and have a crucial role in the initiation of collagen-induced arthritis. Here, we have determined the dependence of T and B cells in collagen-antibody-induced arthritis (CAIA) during different phases of arthritis. Mice deficient for B and/or T cells were susceptible to the CAIA, showing that the antibodies induce arthritis even in the absence of an adaptive immune system. To determine whether CII-reactive T cells could have a role in enhancing arthritis development at the effector level of arthritis pathogenesis, we established a T cell line reactive with CII. This T cell line was oligoclonal and responded to different post-translational forms of the major CII epitope at position 260–270 bound to the A^q ^class II molecule. Importantly, it cross-reacted with the mouse peptide although it is bound with lower affinity to the A^q ^molecule than the corresponding rat peptide. The T cell line could not induce clinical arthritis *per se *in A^q^-expressing mice even if these mice expressed the major heterologous CII epitope in cartilage, as in the transgenic MMC (mutated mouse collagen) mouse. However, a combined treatment with anti-CII monoclonal antibodies and CII-reactive T cells enhanced the progression of severe arthritis.

## Introduction

Collagen-induced arthritis (CIA) is a widely used animal model for rheumatoid arthritis (RA). Immunization with native collagen type II (CII) in adjuvant induces autoimmune polyarthritis in susceptible rodents and primates [1]. The separate roles of T cells and B cells in both the initial and the progression phases of arthritis in this model are still undefined. Clearly, immunization with heterologous CII activates both CII-reactive T cells and B cells. The T cell response is dominated by reactivity to CII used for immunization, and T cells do not readily cross-react with mouse CII [2]. In contrast, B cells produce high levels of autoreactive and arthritogenic IgG antibodies reactive with both heterologous and homologous CII. The most likely scenario is that the heteroreactive T cells give help to autoreactive B cells that cross-react with mouse CII. Molecular identification of the relevant epitopes supports this interpretation because there is a critical difference in the T cell epitope but not in the major B cell epitopes between mouse CII and heterologous CII. Furthermore, depletion of T cells with anti-CD4 or anti-T-cell receptor (anti-TCR) antibodies is more effective if given before immunization than if given afterwards [3,4]. Finally, severe arthritis is readily induced with anti-CII antibodies [5], whereas transfer with T cells induces only synovitis and not clinical arthritis [6].

However, it is unlikely that CIA pathogenesis can be reduced to mediation by anti-CII antibodies alone. The question is whether autoreactive T cells might have an additional role in CIA, in particular whether they have a role in the further progression of arthritis and during the chronic relapsing disease course that follows the initial arthritis in some mouse strains. This possibility has also been highlighted by the finding that many heteroreactive T cells are most probably potentially autoreactive to CII *in vivo*, because a major difference is the binding of the peptide to the MHC rather than interaction with TCR [2,7]. The difference between the mouse and the heterologous immunodominant peptide is dependent on differences in binding to the MHC class II molecule A^q^. Thus, they recognize the same peptide but different densities of the peptide are presented depending on whether the CII is of mouse or of heterologous origin. Interestingly, immunization with mouse CII induces arthritis in a smaller number of mice but gives a more chronic disease course than immunization with heterologous CII [8,9]. Furthermore, in the mutated mouse collagen (MMC) mouse, which expresses a mutated CII with the heterologous CII – namely mutated at position 266, changing Asp to Glu – the heterologous CII is expressed in the joints. In this mouse T cells are partly tolerized and the development of arthritis is differently genetically controlled [10,11].

The development of arthritis after injection of collagen antibodies (collagen-antibody-induced arthritis; CAIA) is thus likely to be different from the development of arthritis in CIA, although the resulting clinical arthritis shares many common characteristics [5]. CAIA is known to develop independently of MHC alleles, whereas CIA is crucially dependent on MHC alleles, with the A^q ^molecule as one of the most susceptible alleles. This suggests that CAIA develops independently of MHC-restricted T cells, and thereby also independently of T cell-dependent B cells. To confirm this assumption directly we investigated mice deficient in B cells and T cells on backgrounds susceptible to CIA. Interestingly, such mice not only developed CAIA but had a more severe disease, suggesting that these cells have a modifying role in this model. We also readdressed the role of transferred T cells by using a T cell line reactive with the major CII epitope 260–270 but with oligoclonal reactivity to the various post-translational modifications. As expected, these T cells could not induce clinical arthritis in either wild-type or MMC mice. However, the transferred T cells enhanced the CII-antibody-induced arthritis into a more prolonged disease course.

## Materials and methods

### Animals

Male B10.Q and QD ([B10.Q × DBA/1]F_1_) mice at 4–6 months of age were used in the present study. The B10.Q strain was obtained from Professor Jan Klein (Tübingen, Germany), and DBA/1 mice were from Jackson Laboratories (Ban Harbor, ME, USA). B cell-deficient mice (μMT mice kindly provided by Dr Werner Muller [Cologne, Germany]) on the (C57Bl/6 × 129)F_1 _background were backcrossed to B10.Q background (12*n*) and T cell-deficient mice (lacking αβ T cells as a result of targeted germline mutation in their TCRβ gene, obtained from Jackson Laboratories) on the (C57Bl/6 × 129)F_1 _background were backcrossed to B10.Q background (6*n*). To obtain mice deficient in both B and T cells, heterozygous female mice deficient in B cells and T cells were crossed with doubly deficient males, and offspring were investigated for the absence of B cells and T cells by cytofluorimetric analysis. Blood cells were stained with anti-CD45Ra (B220 coupled to fluorescein isothiocyanate) and anti-TcR (145-2C11 coupled to phycoerythrin) before analysis. MMC transgenic mice (previously named MMC-1), which originated on the C3H.Q background as described previously [10], were backcrossed for eight generations onto the B10.Q background. The transgene MMC is a mutated mouse CII gene in which position 266 has a been changed from aspartic acid (D) to glutamic acid (E), thereby expressing the rat CII260–270 epitope in a CII-restricted fashion. All mice were kept in a conventional but barrier animal facility (as defined in ) with a climate-controlled environment having 12 hours light/12 hours dark cycles in polystyrene cages containing wood shavings; the mice were fed with standard rodent chow and water *ad libitum*. All animal experiments had been approved by the local animal welfare authorities.

### CII-specific monoclonal antibodies

**Figure 1:**
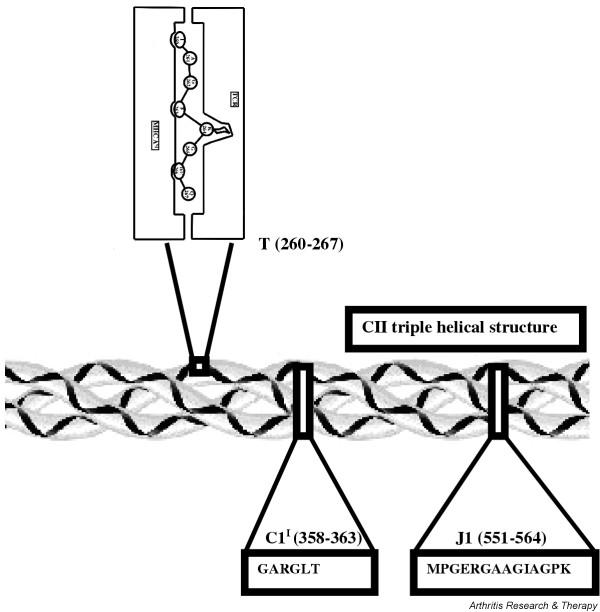
Type II collagen (CII)-specific B and T cell epitopes. Illustration of T (residues 260–267) and B (C1^I^, residues 358–363; J1, residues 551–564) epitopes present in the triple-helical form of the collagen type II recognized by the monoclonal antibodies and the T cells used in this study. As indicated, mouse CII differs from rat CII in position 266. Aspartic acid (D) in mCII is replaced by glutamic acid (E) in rCII. Major post-translational modifications in the CII peptide 260–267 occur in lysine at position 264.

The CII-specific hybridomas were generated and characterized as described in detail elsewhere [12-14]. From the panel of monoclonal antibodies generated, a combination of an IgG2b antibody of the clone M2139 binding to the J1 epitope (amino acids 551–564) and an IgG2a antibody of the clone CIIC1 binding to the C1^I ^epitope (amino acids 358–363) was found to be more arthritogenic [5], whereas CIIF4 monoclonal antibody binding to F4 epitope (amino acids 926–936) was found to be inhibitory [15]. Recent studies *in vitro *also emphasize that these arthritogenic monoclonal antibodies M2139 and CIIC1 suppressed the self-assembly of CII into fibrils, whereas CIIF4 was found to be inert [16]. Figure 1 illustrates the B cell and T cell epitopes present in the CII α-chain recognized by the monoclonal antibodies and the T cell line used in this study. Monoclonal antibodies were generated as culture supernatants and purified by affinity chromatography with γ-bind plus affinity gel matrix (Pharmacia, Uppsala, Sweden). The IgG content was determined by freeze-drying. The antibody solutions were filter-sterilized using syringe filters with a pore size of 0.2 μm (Dynagard; Spectrum Laboratories, CA, USA), aliquoted and stored at – 70°C until use. The amount of endotoxin in the antibody solutions prepared was found to be in the range 0.02–0.08 EU/mg of protein as analysed with the *Limulus *amebocyte lysate (Pyrochrome) method (Cape Cod Inc., Falmouth, MA, USA).

### Passive transfer of antibodies

The cocktail of M2139 and C1 monoclonal antibodies was prepared by mixing equal concentrations of each of the sterile filtered antibody solutions to get a final amount of 9 mg. Mice were injected intravenously twice with 0.25–0.4 ml of antibody solution, with a minimum interval of 3 hours. As a control, groups of mice received equal volumes of PBS. On day 5, lipopolysaccharide (25 μg per mouse) was injected intraperitoneally in all mice. A pair of irrelevant antibodies of the same subclass (mouse anti-human HLA-DRα, IgG2a [L243] and mouse anti-human parathyroid epithelial cells, IgG2b [G11]) did not induce arthritis in the most susceptible strain, BALB/c mice [5].

### Characteristics of CII-specific T cell line

**Figure 2:**
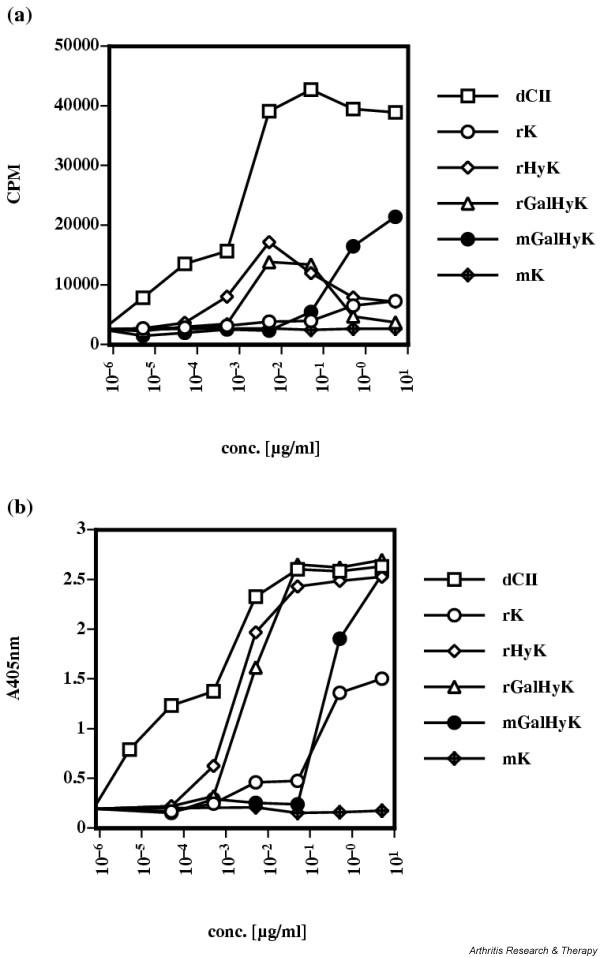
Characteristics of the type II collagen (CII)-specific T cell line. CII-specific T cell line QDHT during passage 5 was used in this study. An aliquot of the cell line used for transfer was tested for proliferation **(a) **and interferon-γ secretion **(b) **after stimulation with different peptides. Mouse and rat CII (mK and rK, respectively), hydroxylated rat CII (rHyK), denatured rat CII (dCII) and mouse and rat galactosylated CII (mGalHyK and rGalHyK, respectively) peptides were used. Rat CII was heat denatured at 50°C for 20 min. Results are representative of several experiments performed with this cell line.

A T cell line specific for rat CII was established as described previously [17]. In brief, draining lymph nodes from rat CII-immunized QD mice (on day 8) were stimulated *in vitro *with rat CII for 4 days. These cells were allowed to rest for a week in the presence of interleukin-2 (IL-2) without antigen-presenting cells. T cells were subsequently re-stimulated with irradiated (3000 rad) syngenic splenocytes and rat CII for 3 days (5 × 10^5 ^T cells/ml, 5 × 10^6 ^antigen-presenting cells/ml, 10 μg/ml rat CII) followed by 2 weeks of resting in medium containing IL-2. At the time of re-stimulation, an aliquot of the cell line was tested for antigen specificity. Lathyritic CII was used for the first *in vitro *re-stimulation, to avoid contamination of pepsin-reactive T cells. The cell line responded towards denatured CII, the non-modified CII 256–270 peptide and the glycosylated CII 256–270 peptide with proliferation and interferon-γ (IFN-γ) production (Fig. 2), but no response towards pepsin was observed. IFN-γ was measured by enzyme-linked immunosorbent assay as described previously [18].

### Arthritis development

Development of clinical arthritis was followed by means of visual scoring of the mice. Mice were examined daily or on alternate days for arthritis development until the end of the experiment. Arthritis was scored with an extended scoring protocol ranging from 1 to 15 for each paw, with a maximum score of 60 per mouse, based on the number of inflamed joints in each paw, inflammation being defined by swelling and redness. Each arthritic toe and knuckle was scored as 1, with a maximum of 10 per paw, and an arthritic ankle or mid-paw was given a score of 5.

### Statistics

Arthritis incidence and severity were analysed by χ^2 ^analysis and the Mann–Whitney *U*-test respectively. *P *≤ 0.05 was considered as significant.

## Results

### Antibody-mediated arthritis in mice deficient in B and/or T cells

**Figure 3:**
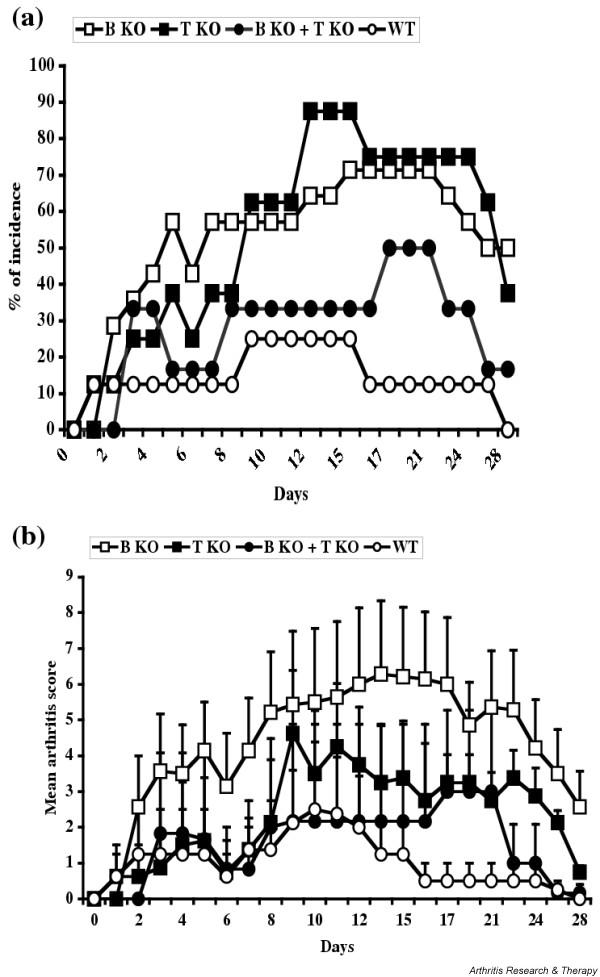
Collagen-antibody-induced arthritis in mice deficient in B cells and T cells. Frequency **(a) **and severity **(b) **of arthritis in groups (*n *= 6–14) of B10.Q mice deficient in B cells (B KO), T cells (T KO), in both B and T cells (B KO+T KO) and wild-type littermate controls (WT). Mice were injected intravenously with 9 mg of monoclonal antibody cocktail on day 0. Another set of animals (*n *= 4–8) from each group were injected with PBS on day 0 as control. All the mice received lipopolysaccharide (25 μg per mouse) intraperitoneally on day 5. All the animals were scored for arthritis up to 1 month and were included in the calculations. None of the control mice developed arthritis. Mice deficient in B cells or T cells were compared with WT mice for statistical calculations. There was no significant difference in arthritis frequency and severity between B cell-deficient and T cell-deficient mice. The error bars in (b) indicate SEM.

To understand the role of B and T cells in antibody-mediated inflammation, mice deficient in either B cells or T cells or both were injected with a combination of two different monoclonal antibodies against CII. The antibodies have been shown to bind to cartilage surfaces shortly after intravenous injection [19] and the epitopes recognized are depicted in Fig. 1. As shown in Fig. 3a, most of the B cell-deficient (71%) and T cell-deficient (87%) mice developed severe arthritis; 50% of the mice deficient in both B and T cells also developed arthritis, whereas only 25% of littermate controls developed the disease (B cell-deficient versus controls, cumulative incidence *P *≤ 0.0354; T cell-deficient versus controls, cumulative incidence *P *≤ 0.0117). Mice deficient in either B or T cells developed more severe arthritis than mice deficient in both populations or than the control littermates (Fig. 3b); however, the difference in arthritis severity between the groups on different days was not significant. These data show that neither T cells nor B cells are necessary for CAIA development. Furthermore, the observed enhancement in the frequency of arthritis in the T cell-deficient mice and the B cell-deficient mice suggest that these cells might play regulatory roles in the initiation of disease.

### Effect of T cells transfer on CAIA

To ascertain whether a transfer of CII-specific T cells after antibody injection induced more susceptibility or prolonged the disease period, we established a rat CII-specific T cell line. The line was established from rat CII-immunized QD mice and re-stimulated *in vitro *four or five times with rat CII before transfer. The established T cell line was A^q^-restricted and oligoclonal because it responded to both the non-modified and hydroxylated, as well as the glycosylated, versions of the major CII peptide 260–270 containing various post-translational modifications at the major T cell recognition site on lysine 264 (Figs 1 and 2). The strongest reactivity was seen to the galactosylated peptide, but the hydroxylated peptide also mounted a strong response. Interestingly, the T cell line cross-reacted to the glycosylated mouse peptide, and the lower reactivity to the mouse glycopeptide than to the rat glycopeptide is most probably dependent on both the lower affinity of the mouse peptide for A^q ^and also a different reactivity of the clonally distinct glycopeptide-reactive T cells [7].

**Figure 4:**
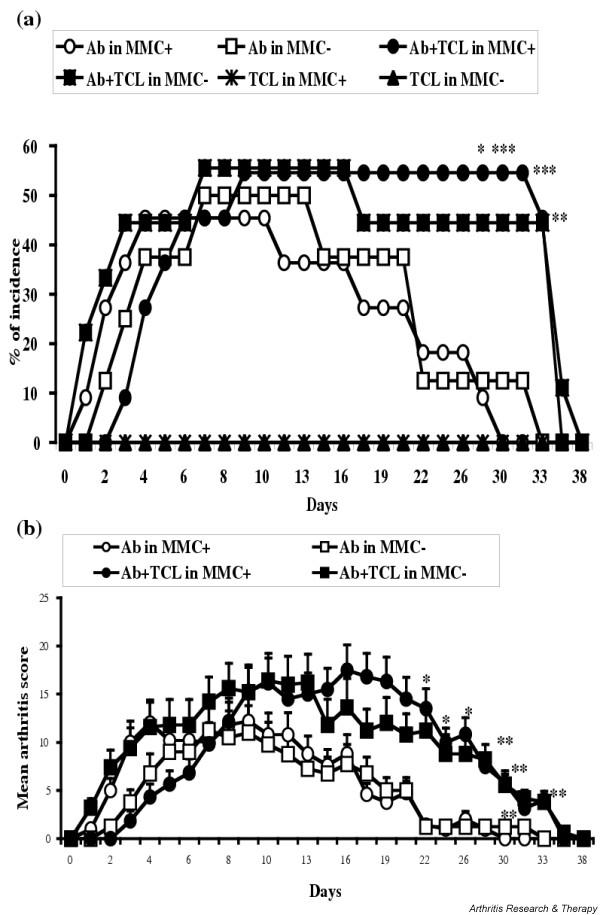
Role of co-transferred type II collagen (CII)-specific T cells in collagen-antibody-induced arthritis. Incidence **(a) **and severity **(b) **of arthritis in different groups of age-matched male mutated mouse collagen (MMC)-positive and MMC-negative littermates (*n *= 7–11) that were injected with either monoclonal antibodies (day 0), T cells (10^7^, day 1) or antibodies and T cells. All mice were monitored for the development of arthritis for 38 days and were included in the calculations. MMC-positive mice injected with antibodies were compared with mice co-transferred with antibodies and T cells. *P ≤ 0.05; **P ≤ 0.01; ***P ≤ 0.005. The error bars in (b) indicate SEM.

To investigate the role of T cells in the acute effector stage of clinical arthritis, newly activated T cells were injected into QD mice intravenously 1 day after the antibody transfer. As expected, injection of the antibodies alone was sufficient to induce arthritis, but co-transfer of T cells did not enhance the initiation of arthritis (Fig. 4). However, transfer of both antibodies and T cells did result in persistent disease activity. As the T cell line was mainly considered as heteroreactive when transferred into wild-type mice (Fig. 2), co-transfer of T cells and antibodies was also performed in MMC mice (which express the rat CII epitope in cartilage) to see whether the presence of truly autoreactive T cells could have a different effect on the acute phase of arthritis. However, T cells again did not affect the initiation phase of the disease; instead, the effect was noted in the more chronic phase of the disease. Still, co-transfer of T cells into MMC mice resulted in an even more pronounced and significant progression of arthritis than in mice that had antibodies transferred. In contrast, wild-type (MMC-negative) mice that had both T cells and antibodies transferred did not show a significant difference from antibody-transferred mice (Fig. 4). Furthermore, an ovalbumin-specific T cell line failed to enhance and perpetuate the arthritis induced by anti-CII antibodies (data not shown).

## Discussion

As we show in the present study, anti-CII monoclonal antibodies are capable of initiating disease independently of B and T cells during the effector phase of arthritis. This is not an unexpected finding because CAIA is induced in naive mice by preformed anti-CII antibodies. Interestingly, however, immune cells might have a regulatory role in CAIA because mice deficient in both T cells and B cells are more susceptible to arthritis than their control littermates. There are several possible explanations for this observation. Clearly, B cells could be regulatory owing to the secretion of a cytokine such as IL-10 [20], the expression of inhibitory receptors such as FcgRIIb [21] or the secretion of regulatory antibodies such as anti-CII antibodies [15,22]. Similarly, there are several ways in which T cells might be regulatory in an effector state like this: for example, they might control bone destruction through interaction with the osteoprotegrin system [23] or through the regulation of cytokines such as IFN-β, tumour necrosis factor-α or IL-4 [24-26]. However, a surprising finding was that mice deficient in both cell types were not as susceptible as the respective single-cell deficient mice. In the doubly deficient mice a complete absence of the adaptive immune response could have led to a more predominant role for the innate immune system in the regulation of the antibody-mediated inflammatory response. In addition, we have shown here that already activated CII-reactive T cells reactive to glycosylated CII could prolong the disease initiated by antibodies, a finding that is highly relevant for comparison with the CIA model.

As in RA, susceptibility to CIA is linked to the expression of certain class II MHC alleles, explaining the crucial role depicted to T cells. The predominant role of T cells in CIA development was demonstrated by using anti-CD4 or anti-TCRαβ monoclonal antibodies and T cell-deficient mice [3,4,27]. Mice deficient in the co-stimulatory molecule CD28 were found to be resistant to CIA [28]. Similarly, administration of CTLA4Ig at the time of immunization prevented the development of CIA [29]. These studies demonstrate the importance of T cell activation in CIA pathogenesis. Depletion of CD4^+ ^T cells has a major influence during the priming phase of arthritis [3] and suppressed the adoptive transfer of disease to severe combined immunodeficient mice using spleen cells from CII-immunized mice [30]. Partial protection of CD4-deficient B10.Q mice and significantly reduced incidence in CD8-deficient mice from CIA suggested an initiating role for the T cells during the priming phase of CIA [31]. However, T cell reactivity alone could not explain the disease pathology in CIA. Transfer of synovitis but not clinical arthritis using CII-specific T cells has previously been shown. In contrast, the high incidence of arthritis induced by native but not denatured collagen indicated the importance of B cells in CIA. It has been shown that mice pre-sensitized with heat-denatured collagen developed progressive arthritis after the transfer of anti-CII antibodies. In addition, adoptive transfer of lymphoid cells together with antibody in the T cell-depleted mice was shown to induce arthritis, and the effector cells were identified as Thy-1^+ ^and L3T4^+^Lyt-2^- ^[32].

The recognition of CII by T cells is critical to the establishment of autoimmune arthritis in CIA. However, it is debatable whether T cells are capable of recognizing tissue antigens such as insoluble CII in the cartilage tissue. It therefore becomes all the more important to understand the role of antigen-specific T cells in arthritis pathogenesis. Antigen-specific T cells might have important roles either during the initiation phase of arthritis or in the perpetuation and exacerbation of the disease after the onset, or they might simply maintain immunity to CII and perpetuate antibody production. Results from the present study demonstrate that CII-specific T cells could have a role in the perpetuation and exacerbation of already established disease rather than having any direct influence on the initiation phase of arthritis.

It is possible that the pro-inflammatory cytokines induced and/or secreted by the co-transferred CII-specific cells could provide a constant cytokine milieu in or near the joints for exacerbating the events induced by the formation of collagen–IgG immune complexes. It should also be noted that the ovalbumin-specific T cell line failed to enhance and perpetuate the arthritis induced by anti-CII antibodies. With the use of CII-specific monoclonal antibodies, it has been shown that IL-1 and tumour necrosis factor-α are the important cytokines for disease development [33], similar to anti-glucose-6-phosphate isomerase antibody-induced disease [34]. The observed enhancement of arthritis in the T cell and B cell singly deficient mice also suggests that these cells might have regulatory roles in the initiation of disease by modulating the cytokine environment. Despite a prolongation of arthritis, co-transfer of the CII-specific T cell line with the monoclonal antibodies did not alter the acute phase of antibody-mediated disease into a chronic disease course, suggesting the importance of other cellular mediators in the pathogenesis of arthritis. However, experiments to understand the factor(s) and cells involved during the progression of arthritis from the initiation stage will provide tools for effective intervention in arthritis progression in patients with RA.

## Conclusions

We demonstrated that anti-CII monoclonal antibodies are capable of initiating arthritis independently of B and T cells during the effector phase of arthritis. Already activated CII-reactive T cells, especially reactive to glycosylated CII, could prolong the disease initiated by antibodies, a finding that is highly relevant for comparison with the CIA model. Experiments to understand the factor(s) and cells involved during the progression of arthritis from the initiation stage could therefore provide tools for effective intervention in arthritis progression in patients with RA.

## Competing interests

None declared.

## Abbreviations

CAIA = collagen-antibody-induced arthritis; CIA = collagen-induced arthritis; CII = type II collagen; IFN-γ = interferon-γ ; IL = interleukin; MMC = mutated mouse collagen; RA = rheumatoid arthritis; TCR = T cell receptor.
